# Histopathological characterization of the oral lichenoid disease subtypes and the relation with the clinical data

**DOI:** 10.4317/medoral.21730

**Published:** 2017-04-08

**Authors:** Javier Alberdi-Navarro, Xabier Marichalar-Mendia, María-José Lartitegui-Sebastián, María-Luisa Gainza-Cirauqui, María-Ángeles Echebarria-Goikouria, José-Manuel Aguirre-Urizar

**Affiliations:** 1 Oral Medicine and Oral and Maxillofacial Pathology Units, Dental Clinic Service. Department of Stomatology II. UFI 11/25. University of the Basque Country/ EHU; 2 Department of Dental Surgery, Faculty of Dental Surgery, University of Malta

## Abstract

**Background:**

The aim of the study was to analyze the histopathological characteristics of samples with a diagnosis of oral lichenoid disease (OLD) and their link with the location and the type of clinical lesion, and the clinicopathological subtypes.

**Material and Methods:**

Retrospective study on 85 consecutive patients diagnosed with OLD (58 women and 27 men, mean age of 57.7 years). Clinical and histopathological characterization of each case (modified WHO criteria). Collection of the clinical and histopathological data of the lesions. Descriptive and comparative statistical analysis of the results.

**Results:**

The 78.8% of the cases were considered clinically typical while the 21.2% were considered compatible. Histologically, 52.9% were classified as typical and 47.1% as compatible. Biopsies from “plaque-like” lesions presented hyperkeratosis (*p*<0.001) and epithelial dysplasia (*p*=0.06) more frequently. Furthermore, acute inflammation was more evident in erosive-ulcerative lesions (*p*=0.001). Differences regarding the location of the biopsy were statistically non-significant. However, 42.9% of the tongue biopsies showed epithelial dysplasia.

**Conclusions:**

The histopathological aspect of this disorder is not specific and does not allow us to differentiate between the main subtypes. Therefore, the main reasons to perform a biopsy in this disorder are to define the differential diagnosis and to rule out epithelial dysplasia or a carcinoma. The final histopathological result may be subject to the type of lesion that is biopsied.

** Key words:**Oral lichenoid disease, histopathology, subtypes, characterization, oral lichen planus, oral lichenoid lesion, epithelial dysplasia.

## Introduction

The term oral lichenoid disease (OLD) encompasses a group of pathological processes with an immunological base, generically called “oral lichen planus”, and that present characteristically with white papules on the oral mucosa (1). The etiology of this disease is unknown in most cases and is considered an oral potentially malignant disorder, with major controversies on its classification, its clinical and histological diagnosis, and its prognosis (1-6).

Based on previous studies (2,7) that showed important prognostic implications, we consider that there are two main OLD sub-types: oral lichen planus (OLP) and oral lichenoid lesions (OLL) (1,7-9).

Typically, the clinical and histopathological assessments are required in all cases in order to perform a proper diagnosis of these disorders (6,8,9). An adequate clinical and histopathological analysis is crucial to reach a diagnosis and to assess the malignant potential of each case (2,6).

Most studies (2,7,10,11) agree, that the application of the WHO diagnostic criteria, modified by van der Meij and van der Waal in 2003 (8) for lichenoid processes, has proven to be inefficient in establishing a good correlation between the clinical and histopathological diagnosis, and to differentiate between oral lichen planus (OLP) and oral lichenoid lesions (OLL). Nonetheless, the study by Rad *et al.* in 2009 (12), showed a good correlation between the clinical and the histopathological aspects.

Classically, several histopathological data have been considered as the differentiating characteristics between lichen planus and lichenoid lesions, such as a deeper inflammatory infiltrate, perivascular inflammation, inflammatory cells such as polymorphonuclear neutrophils, etc. (6,13,14). Moreover, the presence of epithelial dysplasia is a controversial datum in the histopathological analysis of these biopsies and, for some authors (6,8), it may even invalidate the diagnosis of oral lichen planus.

The lack of clear histopathological criteria that allow us to differentiate the main subtypes has motivated some to question the need to perform a diagnostic biopsy on this disorder (15). For these authors (15), the diagnostic biopsy would not be strictly nec-essary, although performing it would be a cautious measure in order to assess the presence or absence of epithelial dysplasia (16). Other authors support this premise (5,16,17).

The relationship between the histopathological aspect of this disorder and some clinical parameters, such as the location of the lesions and the type of clinical lesion biopsied, has been sparsely studied (18,19). Recently, however, these clinical parameters have been suggested as possible modifying factors of the histopathological aspects (6).

Based on this data, we designed this study to determine the histopathological characteristics of the biopsies of this disorder and its main subtypes, and to determine the relation with the clinical data of location and type of lesion biopsied.

## Material and Methods

We performed a retrospective study on 85 patients clinically diagnosed with OLD and who had a diagnostic biopsy from the oral mucosa involved. The study was performed in the Oral Medicine and Oral and Maxillofacial Pathology Units of the Dental Clinic Service of the University of the Basque Country/ EHU.

The group of patients comprised 58 (68.2%) women and 27 (31.8%) men, with a mean age of 57.7 years at the time when the biopsy was taken (range 34-91).

This study was approved by the Ethics, Investigation and Teaching Committee (CEISH) of the University of the Basque Country/ EHU (CEISH185/2012).

A specific clinicopathological diagnostic protocol was complied for all cases following the criteria established by van der Meij and van der Waal (8) and Cortés *et al.* (9). Based on these criteria, patients and biopsies were classified as follows: Clinically Typical (CT), Clinically Compatible (CC), Histopathologically Typical (HT) and Histopathologically Compatible (HC).

To consider a case as CT, the patient had to comply with the following conditions: 1. Presence of bilateral and roughly symmetrical lesions, 2. Presence of white-grey papules in a reticular pattern, 3. Presence, occasionally, of erosive-ulcerative, vesicular and/or plaque-like lesions. When any of these characteristics was absent, it was considered a CC case (8,9).

Regarding the histopathological features, a case was considered HT when the following characteristics were fulfilled: 1. Presence of a “band-like” chronic inflammatory infiltrate (mainly lymphocytic), 2. Presence of hydropic degeneration of the basal layer, and 3. Absence of dysplasia. When any of these characteristics was absent, the case was considered as HC (8,9).

Three specialists in Oral Pathology performed the histopathological assessment on H&E samples by gathering and assessing the main histopathological characteristics that differentiate between oral lichen planus and oral lichenoid lesions (13,14). Agreement was reached on the results with a Kappa index of 0.84.

A descriptive and comparative statistical analysis was performed with the data obtained (Chi-square test). Results were considered statistically significant when *p* ≤ 0.05.

## Results

Following the criteria established by van der Meij and van der Waal in 2003 (8) and Cortés *et al.* in 2009 (9), 67 (78.8%) of the patients were classified as Clinically Typical and 18 (21.2%) as Clinically Compatible. Histopathologically, 40 (47.1%) of the biopsies from our patients were classified as Histopathologically Typical and 45 (52.9%) as Histopathologically Compatible.  Table 1 shows the key data of these groups.

**Table 1:**
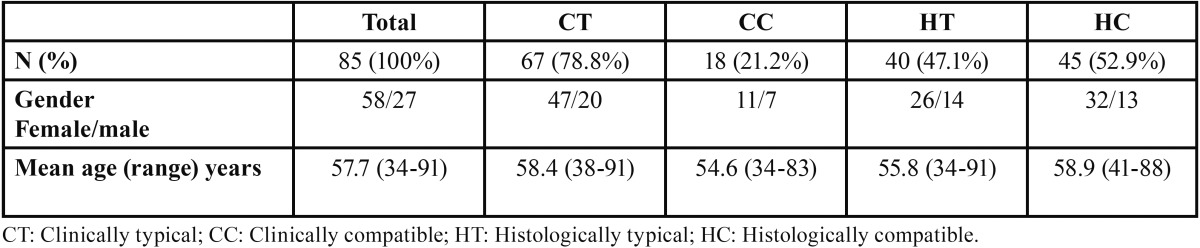
Clinical and histopathological classification of the sample according to the subtype (van der Meij and van der Waal 2003; Cortés *et al.* 2009) (8,9).

The histopathological characteristics of the biopsies in relation to the clinical subtypes are shown in  Table 2. Differences in all the studied data were non-significant.

**Table 2:**
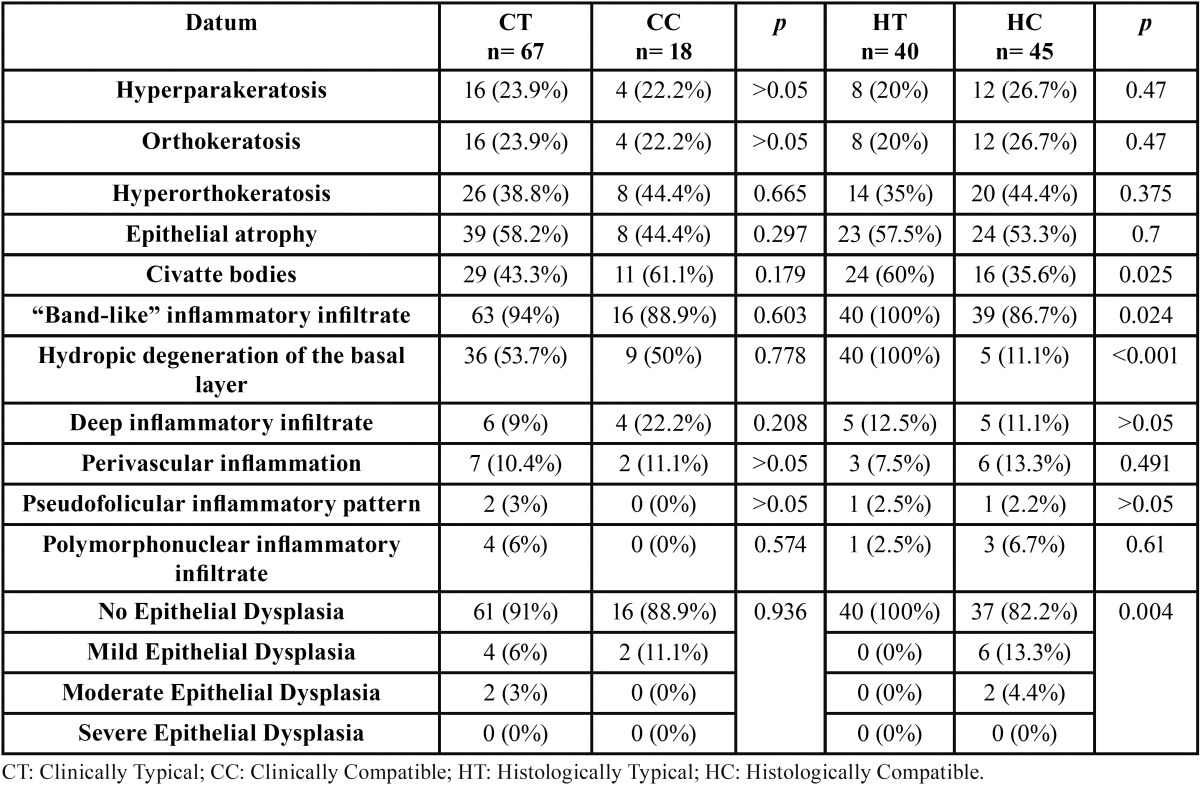
Main histopathological characteristics of the clinical and histological subtypes of OLD.

**Figure 1:**
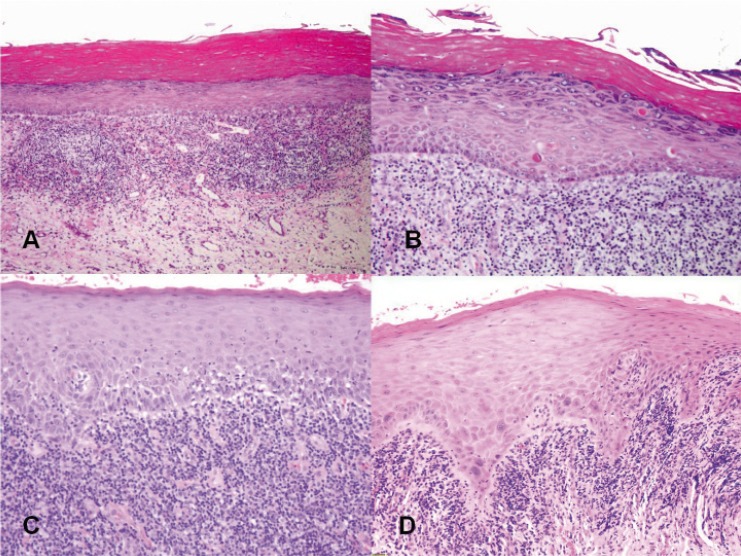
Histopathological data from the Oral Lichenoid Disease (OLD) biopsies. A) Hyperorthokeratosis, flattening of the ridges and “band-like” chronic lymphocytic inflammatory infiltrate. H&E 20x. B) Hyperorthokeratosis with granulosis, Civatte bodies and chorionic inflammatory infiltrate. H&E 40x. C) Basal layer degeneration, lymphocytic inflammatory infiltrate and vascular proliferation. H&E 40x. D) Mild dysplastic changes and chronic lymphocytic inflammatory infiltrate. H&E 40x.

 Table 2 shows the histopathological characteristics of the biopsies in relation to the histopathological subtypes (Fig. 1). A statistically significant higher number of Civatte bodies was observed in the HT group (*p*=0.025).

Regarding the location of the biopsy, 62 (72%) cases had a biopsy of the buccal mucosa, making it the most frequently biopsied, followed by the gingiva in 13 (15.3%) cases, the tongue in 7 (8.2%), the palate in 2 (2.3%) and the floor of the mouth in 1 (1.2%) case. The last two locations were grouped as “others”. Non-significant differences were observed between the different locations and the histopathological aspects. Nonetheless, the 42.9% of the biopsies performed in tongue showed epithelial dysplasia (*p*=0.089) ( Table 3).

**Table 3:**
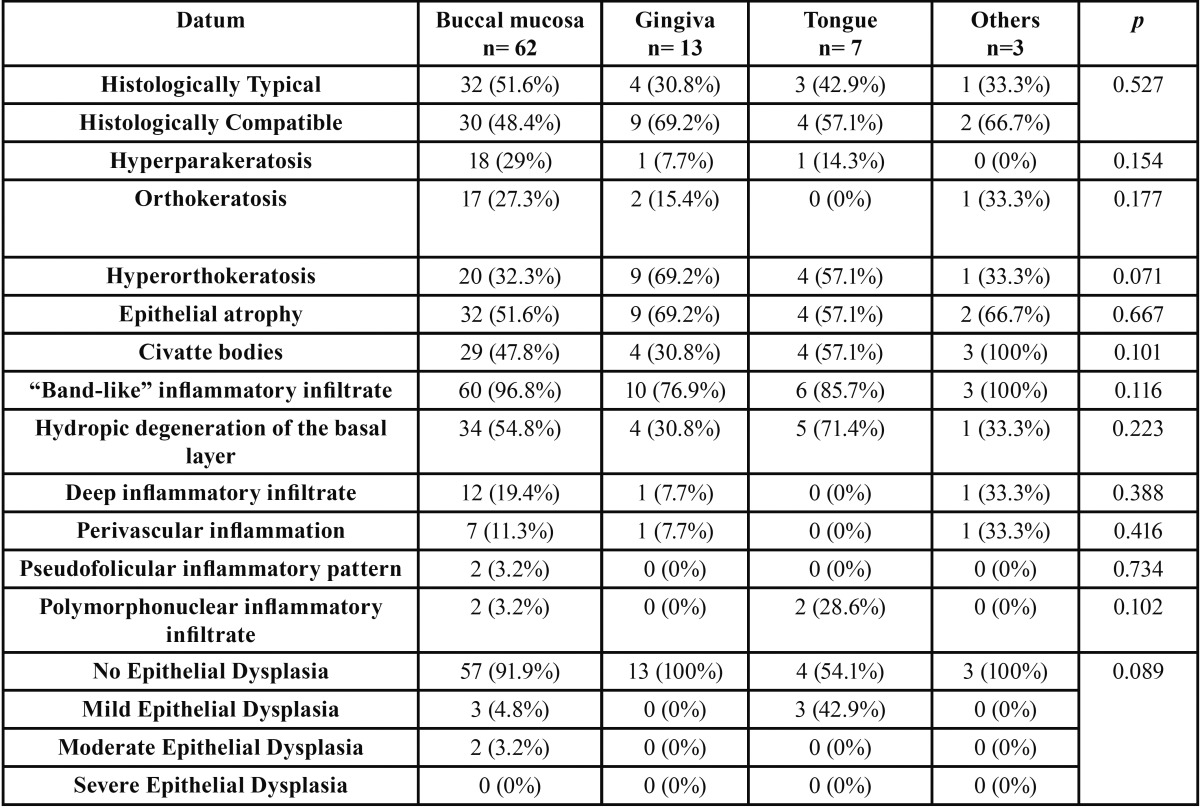
Histopathological characteristics of the OLD biopsies in relation to the location of the biopsied lesion.

When considering the type of lesion biopsied, the most frequent presentation were the papules in 55 (64.7%) cases, followed by the erosive-ulcerative lesions in 17 (20%) cases and the plaque lesions in 13 (15.3%) cases. We recognized a higher frequency of hyperkeratosis (*p*=0.001) and epithelial dysplasia (*p*=0.006) in the plaque lesions. Furthermore, we observed greater presence of acute inflammatory infiltrate in cases of erosive-ulcerative lesions (*p*=0,001). Although the differences were non-significant, we recognized a higher prevalence of deep inflammatory infiltrate in non-papular lesions (*p*=0.058) ( Table 4).

**Table 4:**
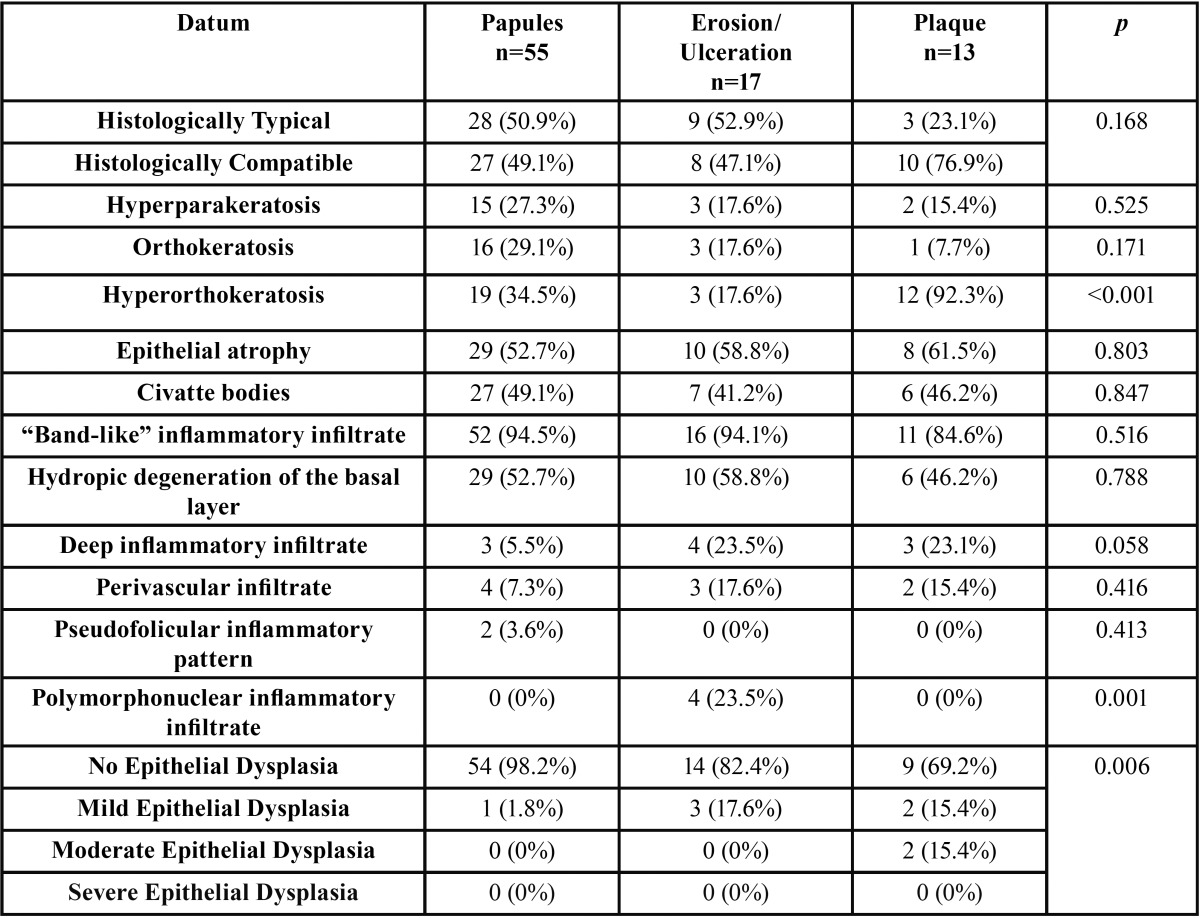
Histopathological characteristics in relation to the type of lesion biopsied.

## Discussion

“Oral lichenoid disease” continues to be a controversial oral potentially malignant disorder that encompasses different processes of an immunological base presenting clinically as white papules in the oral mucosa (1). Performing an adequate diagnosis is essential to determine the true ability of a malignant transformation, considered to be different depending if it is a typical case (OLP) or a compatible case (OLL) (2,7).

Our results confirm that there is no relation between the different clinical and histopathological subtypes of this disease, as has been presented in other studies (2,7,10,11). Therefore, we consider that the histopathological characteristics observed in the biopsies of this disorder should not be used as infallible and specific diagnostic criteria. A reasonable explanation would be considering the natural course of this pathology, chronic and dynamic; therefore, the histopathological aspect may vary, depending on the biological moment in which this biopsy is performed (6,20,21). Another key aspect would be related to the location and the type of lesion of the biopsy (6).

Few studies (18,19) have evaluated the clinical aspect in relation to the histopathological characteristics of the biopsies in these patients.

Karatsaidis *et al.* (18), compared the epithelial thickness in papular and erythematous lesions showing a greater thinning of the epithelium in the later, as would have been expected. Furthermore, Fernández-González *et al.* (19) assessed histopathological aspects in relation to clinical aspects. They document a greater keratinization in biopsies from reticulo-papular lesions and the presence of polymorphonuclear neutrophils in biopsies from atrophic-erosive lesions.

In our study, the histopathology showed non-significant differences in relation to the location of the lesion from where the biopsy was taken. We did, however, recognize a greater presence of hyperorthokeratosis in biopsies taken from the gingiva and the tongue that we believe may be due to a physiological factor as these locations are formed by masticatory mucosa, which would have a greater tendency to form orthokeratosis.

A result to highlight from our study is that we observed epithelial dysplasia in 42.9% of the biopsies performed on the tongue although, in most cases, it was mild. Taking this finding into account, as well as considering that one of the aims of the histopathological study in patients with this disorder is to rule out epithelial dysplasia (15), and that the tongue is the most common location for oral squamous cell carcinoma (22), we believe that this should be the location of choice when performing a biopsy in this disorder.

The presence of epithelial dysplasia is an important and controversial aspect in the histopathological analysis of these biopsies. In the recent years, several articles have referred to epithelial dysplasia as a clinical process by using the term “oral dysplasia” (6,23). We consider this as a misconception since epithelial dysplasia is a histopathological concept that is characterized by a group of architectural and cytological morphological alterations and not a clinical condition (24).

Furthermore, as other authors have stated (18), our group considers that the term “lichenoid dysplasia” devised by Krutchkoff and Eisenberg in 1985 (25) is inappropriate, since it can lead to confusion and can be misinterpreted by the clinician or by the surgeon. The chronic inflammatory infiltrate associated to lesions with epithelial dysplasia is an immunological activation phenomenon associated with a carcinogenic event and its origin is different from the origin of the infiltrate observed in oral lichenoid disease (26,27).

One of the main characteristics of this disorder is the presence of a “band-like” parabasal chronic inflammatory infiltrate that, together with the hydropic degeneration of the basal layer, defines the “interphase or lichenoid mucositis” (5).

The polymorphonuclear infiltrate observed in these biopsies, has been typically linked with the OLL cases (14). Nevertheless, our results show that the type of lesion biopsied conditions this infiltrate in great measure, as pointed out by Fernández-González *et al.* (19). In our case, this polymorphonuclear inflammatory infiltrate was observed only in erosive-ulcerative lesions. Furthermore, the study by Thornill *et al.* (14) failed to consider the type of lesion that was biopsied.

In recent years, controversy on the existence and relevance of plaque-like lesions in patients with OLD has emerged, even pointing out that it may anticipate a proliferative verrucous leukoplakia (PVL) (28-30). In relation to this, we should mention that, in our study, epithelial dysplasia was more significant in biopsies from plaque-like lesions. This result would support the importance of always performing a biopsy when these lesions are present in OLD patients.

Finally, in relation to the subtypes in this disorder (typical/compatible), the only significant histopathological difference observed was on the higher number of Civatte bodies present in the histopathologically typical cases. This finding probably reflects a greater presence of hydropic degeneration of the basal layer of the epithelium associated with an evident lymphocytic inflammatory infiltrate in the chorion, all of which characterize the histopathologically typical cases.

With our findings, we consider that we are incapable of differentiating between the subtypes of OLD with the histopathological data. Therefore, we believe that the main aims in performing a biopsy in this disorder should be to differentiate it from other specific pathologies of the oral mucosa and to rule out the epithelial dysplasia or an oral squamous cell carcinoma.

We believe that, when performing a biopsy, the location and type of clinical lesion are very important aspects to assess as it can modify, in great measure, the histopathological aspects.

As a practical conclusion from our study, we consider that, when possible, a biopsy of the lesions of the tongue and of the plaque-like lesions should be performed since these have shown a higher percentage of epithelial dysplasia and, therefore, would have a higher risk of malignant transformation in this oral premalignant disorder.
